# Anti-GAD65 Containing Cerebrospinal Fluid Does not Alter GABAergic Transmission

**DOI:** 10.3389/fncel.2016.00130

**Published:** 2016-05-18

**Authors:** Jana K. Hackert, Lorenz Müller, Marco Rohde, Christian G. Bien, Rüdiger Köhling, Timo Kirschstein

**Affiliations:** ^1^Oscar Langendorff Institute of Physiology, University of RostockRostock, Germany; ^2^Epilepsy-Center Bethel, Krankenhaus MaraBielefeld, Germany

**Keywords:** GAD65-antibodies, GABA receptor, autoimmune encephalitis, CSF, IPSC, IPSP

## Abstract

Glutamic acid decarboxylase of 65 kDa (GAD65) antibodies have been reported in a variety of neurological disorders such as stiff-person syndrome (SPS), sporadic ataxia and some cases of epilepsy. Since the target is believed to be the cytoplasmic enzyme GAD65, the key enzyme of γ-aminobutyric acid (GABA) synthesis, the pathophysiological role of these antibodies is poorly understood. Here, we stereotactically injected human cerebrospinal fluid (CSF) containing GAD65-antibodies into the hippocampus of rats *in vivo* and then prepared hippocampal slices 1–2 days after post-operative recovery. We characterized both evoked and spontaneous GABAergic transmission *in vitro* using sharp microelectrode and patch-clamp recordings in CA1 neurons. Intracellular recordings with sharp microelectrodes from CA1 neurons showed that evoked GABA_A_R- or GABA_B_R-mediated inhibitory postsynaptic potentials (IPSP) remained unaltered in anti-GAD65 tissue. These results were confirmed with patch-clamp recordings showing no difference in evoked gabazine-sensitive inhibitory postsynaptic currents (IPSCs). In addition, spontaneous IPSCs also showed no difference between anti-GAD65 tissue and controls with respect to the mean frequency, the mean amplitude and the sIPSC distribution. In conclusion, stereotactic injection of GAD65-antibodies into the hippocampus leaves evoked and spontaneous GABAergic synaptic transmission intact. Hence, dysfunction of the inhibitory GABAergic system does not appear to be the major mechanism of epileptogenicity in this disease.

## Introduction

Autoimmune encephalitis is increasingly recognized in patients with otherwise unexplained temporal lobe epilepsy. Immune-mediated encephalitides were usually considered to be paraneoplastic in origin, first described in 1960 (Brierley et al., 1960), and many reported cases were associated with specific autoantibodies—mainly to Hu (anti-neuronal nuclear antibody-1, ANNA-1), in patients with lung cancer (Dalmau et al., 1992; Alamowitch et al., 1997; Graus et al., 2001), to Ma2 (paraneoplastic antigen Ma2) in patients with testicular tumors (Voltz et al., 1999), to CRMP5/CV2 (collapsin response mediator protein 5) in patients with thymomas (Antoine et al., 1995) or to NMDA receptors in patients with ovarian teratomas (Dalmau et al., 2007). In addition, cases of non-paraneoplastic autoimmune encephalitis have been described (Bien et al., 2000; Mori et al., 2002), associated with systemic autoimmune disorders, autoantibodies against cell membrane antigens such as voltage-gated potassium channels (Buckley et al., 2001; Schott et al., 2003; Vincent et al., 2004) later refined as antibodies to elements of the potassium channel complex, namely leucine-rich, glioma inactivated 1 (LGI1) and contactin-associated protein 2 (CASPR2; Irani et al., 2010; Lai et al., 2010; Lancaster et al., 2011), and autoantibodies against intracellular antigens such as glutamic acid decarboxylase (GAD; Saiz et al., 2008; Malter et al., 2010).

GAD-antibodies were initially identified in the serum and cerebrospinal fluid (CSF) of patients with stiff-person syndrome (SPS; Solimena et al., 1988). This syndrome often occurs in association with other autoimmune diseases, mainly type 1 diabetes mellitus (DM1; Baekkeskov et al., 1990). GAD-antibodies are present in about 80% of newly diagnosed DM1 patients, although the levels are usually 100-fold higher in SPS than in DM1 (Meinck et al., 2001). GAD-antibodies were also reported in a subgroup of patients with late-onset isolated cerebellar ataxia (Honnorat et al., 2001), and in the last years more frequently in patients with limbic encephalitis and epilepsy (Saiz et al., 2008). GAD-antibodies may be found in patients with epilepsy in two different settings (Bien and Scheffer, 2011): (i) acute/subacute onset of seizures accompanied by variable degrees of cognitive and psychiatric disturbance, typically in association with MRI evidence of inflammation of mesial temporal structures (limbic encephalitis; Malter et al., 2010); and (ii) in patients with chronic epilepsy without clinical or MRI evidence of active CNS inflammation (Liimatainen et al., 2010).

As the primary target is believed to be GAD65 which is a cytoplasmic enzyme, the pathophysiological role of GAD-antibodies is still unresolved (in many patients, the antibodies are also directed to GAD67; isolated GAD67 antibodies are a very rare exception). GAD is the rate-limiting enzyme for γ-aminobutyric acid (GABA) synthesis that catalyzes the conversion of glutamic acid to the inhibitory neurotransmitter GABA, and is diffusely present in the central nervous system (CNS; Lernmark, 1996). One attractive hypothesis of the pathophysiological role of GAD-antibodies is the inhibition of synaptic GAD activity, leading to a decrease in GABA synthesis in nerve terminals; an alternative assumption is interference with GABA release (Dinkel et al., 1998; Mitoma et al., 2000). Hence, altered GABAergic transmission could lead to increased neuronal excitability and lower seizure threshold.

It is not known whether GAD65-antibodies play a pathophysiologically relevant role *in vivo*. Therefore, we performed stereotactic injection of CSF containing GAD65-antibodies into the rat hippocampus in order to study GABAergic transmission in the CA1 region of hippocampal slices. We found that GAD65-antibodies applied *in vivo* did not alter GABAergic synaptic transmission in hippocampal slices.

## Materials and Methods

### Stereotactic Intrahippocampal Injection *In Vivo*

Bilateral stereotactic injection of CSF samples into the hippocampus was performed in 2-month-old female Wistar rats (190–260 g, Charles River, Sulzfeld, Germany). CSF samples containing high-titer GAD65-antibodies were obtained from three patients with focal epilepsy due to limbic encephalitis (all female; 18, 27 and 50 years old). GAD65-antibodies were detected by indirect immune-techniques by congruent GAD65 specific signals on mouse brain, transfected cells and immune-dot-blot (all from Euroimmun, Lübeck, Germany). Titration was done on cell based assays in dilution steps of 1:2 and multiples. All titers were higher than 1:250. All patients gave informed consent to use the CSF samples for scientific purposes. For control experiments, sterile saline was used for stereotactic injection. All animal procedures were approved by the local Animal Welfare committee and conformed to national and international guidelines on the ethical use of animals (European Council Directive 86/609/EEC, approved by the Regional Authorities of Agriculture, Food Safety and Fishery Mecklenburg-West Pomerania, No. 7221.3–1.1–017/11). All efforts were made to minimize animal suffering and to reduce the number of animals used.

Surgery was performed in animals anesthetized with S-ketamine (100 mg/kg i.p.) and xylazine (15 mg/kg i.p.) and mounted on a stereotactic frame (Narishige, Tokyo, Japan). The injection site (relative to bregma) was optimized using ink injection and two sets of coordinates were used for animals < 220 g (5.2 mm posterior, ±4.3 mm lateral, 4.8 mm deep) and animals > 220 g (4.7 mm posterior, ±4.5 mm lateral, 6.0 mm deep). The injection of 5 μl of native non-diluted CSF in 10 steps of 0.5 μl every 2 min into both hippocampi was performed with a Hamilton syringe (75 N; Hamilton AG, Bonaduz, Switzerland). Following the last 0.5-μl-step, the syringe was left *in situ* for another 2 min to optimize CSF intrusion into the brain parenchyma. After surgery, the rats recovered quickly and were sacrificed 1–2 days following stereotactic injection.

### Hippocampal Slice Preparation

After deep anesthesia with diethyl ether (Mallinckrodt Baker, Deventer, Netherlands), rats were decapitated, and the brains were dissected out quickly and submerged into oxygenated iced sucrose-based dissection fluid containing (in mM) NaCl 87, sucrose 75, KCl 2.5, NaHCO_3_ 25, NaH_2_PO_4_ 1.25, CaCl_2_ 0.5, MgCl_2_ 7, and glucose 10, pH 7.4, osmolarity 300–310 mosmol/kg H_2_O. Transversal horizontal brain slices (400 μm) of the hippocampus were prepared using a vibratome (Integraslice 7550MM, Campden Instruments, Loughborough, UK), and then transferred into a storage chamber containing sucrose-based dissection solution. Slices were continuously gassed with 95% O_2_ and 5% CO_2_ to maintain the pH at 7.4 and allowed to recover at room temperature for at least 1 h before being placed into an interface chamber (BSC HT, Harvard Apparatus, Holliston, USA) perfused with standard artificial cerebrospinal fluid (ACSF) containing (in mM) NaCl 125, KCl 3, NaHCO_3_ 21, NaH_2_PO_4_ 1.25, CaCl_2_ 2.5, MgCl_2_ 1.0 and glucose 13, pH 7.4, osmolarity 295–305 mosmol/kg H_2_O (2 ml/min). The recording temperature was maintained at 32°C (TC-10, npi electronic, Tamm, Germany).

### Intracellular Recordings

Sharp microelectrode recordings were used to study GABA_A_-receptor and GABA_B_-receptor-mediated inhibitory postsynaptic potentials (IPSPs). To this end, CA1 pyramidal cells were impaled with a borosilicate glass microelectrode (60–130 MΩ, pulled with P-97, Sutter Instrument, Novato, USA and filled with 3 M potassium acetate and 0.3 M KCl) by using an SEC-10L amplifier (npi electronic). A unipolar stimulation electrode placed into the CA1 region, nearby the recording electrode, was used to stimulate adjacent interneurons. GABA-mediated IPSPs were isolated pharmacologically in the presence of the NMDA-receptor blocker D-AP5 (50 μM) and the AMPA receptor blocker 6-cyano-7-nitroquinoxaline-2, 3-dione disodium (CNQX, 10 μM, both from Tocris). Under these conditions, resting membrane potential, membrane resistance, and membrane time constant were determined. Membrane resistance was calculated as the slope of the steady-state current-voltage relationship obtained by hyperpolarizing current injection (ranging from −1.4 to +1.4 nA in 100-pA-steps for 600 ms). Membrane time constant was calculated as the average time constant during the hyperpolarizing steps. Increasing stimulation strength (from 20 to 400 μA in 20-μA-steps every 20 s) applied to interneurons evoked increasing amplitudes of GABA_A_R- and GABA_B_R- mediated IPSPs.

### Patch-Clamp Recordings

Patch-clamp recordings of GABA_A_ receptor-mediated inhibitory postsynaptic currents (IPSCs) were performed to obtain kinetic data of GABAergic transmission. Recordings from CA1 pyramidal cells were performed in the whole-cell mode with recording electrodes (4–8 MΩ) filled with internal solution containing (in mM) CsCl 145, HEPES 20, NaCl 2, Mg-ATP 2, GTP 0.3, KOH-EGTA 0.2 (pH 7.2, adjusted with KOH; 310 mosmol/kg H_2_O). QX-314 (5 mM) added to the internal solution prevented the cells from voltage-dependent spiking. In order to isolate GABAergic currents, the NMDA-receptor blocker D-AP5 (50 μM) and the AMPA receptor blocker CNQX (10 μM, both from Tocris Bioscience, Bristol, UK) were added to the ACSF before the patch pipette was lowered to the slice. The stimulation electrode was placed nearby the recording electrode to activate interneurons. GABAergic currents were recorded at room temperature (24°C) in voltage-clamp mode (holding potential −50 mV) with a patch-clamp amplifier (ELC 03 × S, npi), low-pass filtered at 2 kHz, digitized at 10 kHz (Micro1401, CED, Cambridge Electronic Design, Cambridge, UK) and analyzed with Signal 2.16 (CED). Series resistance was monitored, and cells were excluded from analysis when series changes in series resistance exceeded 100% or 30 MΩ. Increasing stimulation strength (from 0.05 to 0.5 mA in 0.05-mA-steps every 10 s) applied to interneurons evoked increasing amplitudes of GABA_A_R-mediated IPSCs. In addition, cells were held at varying membrane potentials (from −50 to +20 mV in 10-mV-steps) during stimulation in order to measure the variation coefficient and the reversal potential. In order to measure time constants for rise and decay (τ_rise_ and τ_decay_), latency and charge (area under the curve), we averaged 25 GABA_A_R-mediated IPSCs at half-maximum amplitude. To assess the duration of the IPSC, we determined the width at 10% of peak amplitude. Variability in GABA_A_R-mediated IPSCs was expressed as the coefficient of variation (CV), defined as the ratio of the standard error to the mean of the IPSC amplitudes. Membrane resistance was calculated as the slope of the steady-state voltage-current relationship obtained by hyperpolarization (ranging from −60 to −90 mV in 5-mV-steps for 300 ms). Finally, gabazine (5 μM) was administered to confirm GABA_A_-receptor dependence.

### Data Processing and Statistical Analysis

Digitization and offline data processing were performed with the CED package (Micro1401 analog-to-digital converter, Signal 2.16 Software, all from CED). All data are expressed as means ± standard error of the mean (SEM). Student’s *t* test or analysis of variance (ANOVA) were used to test for statistical significance. The level of significance is set to 0.05.

## Results

### Evoked GABAergic Transmission is Intact in Anti-GAD65 Tissue

**Figure 1:**
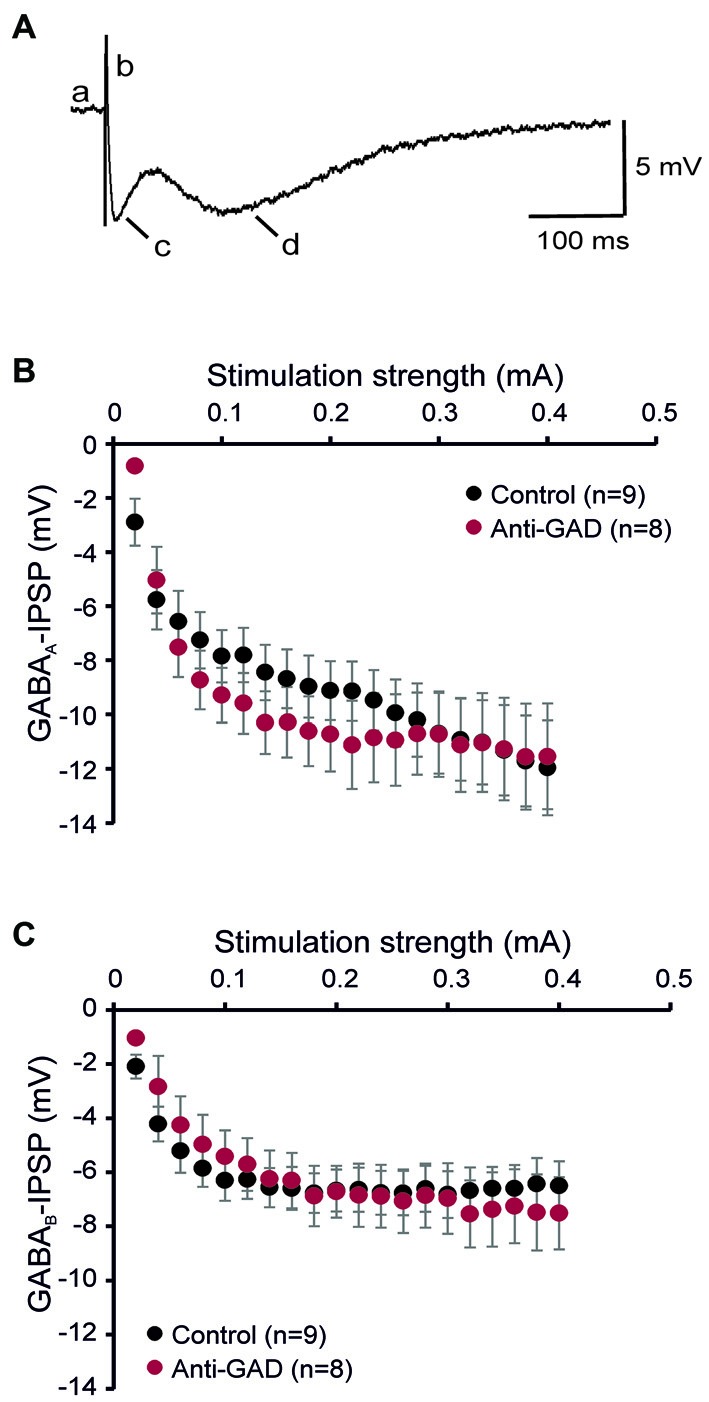
**GABA_A_ and GABA_B_ receptor-mediated inhibitory postsynaptic potentials (IPSPs) are intact in anti-GAD65 tissue. (A)** Sample trace of an intracellular recording evoked by increased near field stimulation showing the resting membrane potential (a), the stimulus artifact (b), the fast GABA_A_R-mediated IPSP component (c) and the slow GABA_B_R-mediated IPSP component (d). **(B)** The input-output relationship of the GABA_A_R-mediated IPSP component did not reveal differences between anti-GAD65-treated animals and controls. **(C)** The input-output curve of the GABA_B_R-mediated IPSP component was also not different between anti-GAD65-injected and control animals.

To study the role of GAD65-antibodies in evoked GABAergic transmission, we stereotactically injected GAD65 antibodies into the hippocampus *in vivo*, and then recorded IPSP in slices prepared 1–2 days after surgery using sharp microelectrode recordings from CA1 pyramidal cells. GABA receptor-mediated IPSPs in CA1 neurons were evoked by focal stimulation applied at increasing intensities (20–400 μA in 20-μA-steps) under blockade of glutamatergic transmission (CNQX and D-AP5 to block AMPA and NMDA receptors, respectively). GABA_A_R- and GABA_B_R-mediated components were distinguished by their kinetics (see lowercase letters in Figure 1A). In five cells, we confirmed gabazine sensitivity (1 μM) of the first component (indicated by “c” in Figure 1A) demonstrating GABA_A_R-dependence (Anti-GAD: −0.2 ± 0.0 mV, *n* = 2; Control: −0.4 ± 0.2 mV, *n* = 3). Input-output relationships of GABA_A_R- and GABA_B_R-mediated IPSPs from rats injected with anti-GAD65-CSF vs. control were not significantly different (Figures 1B,C). These results show that *in vivo* stereotactic injection of GAD65-antibodies into the hippocampus does not cause any depression of GABAergic synaptic transmission *in vitro*. Importantly, also the resting membrane potential observed in these intracellular recordings was almost identical in anti-GAD65-treated and control tissues (Table 1). However, there was a tendency towards higher membrane resistance in cells from anti-GAD65-treated animals that might hint at a mildly impaired tonic GABAergic steady-state conductance in these cells. By contrast, experiments with gabazine to block GABA_A_R IPSP components showed that the gabazine-induced depolarization was stronger in cells from anti-GAD65-treated tissue than in control cells as assessed by amplitude of current injection necessary to hold a membrane potential of −70 mV (Anti-GAD: −144 ± 10 pA, *n* = 2; Control: −51 ± 2 pA, *n* = 3, *P* < 0.05), which in turn argues against impaired tonic GABA inhibition. Thus, we conclude from our sharp microelectrode data that evoked GABAergic inhibition in anti-GAD65-treated tissue is left intact.

**Table 1:** **CA1 pyramidal cell properties (intracellular recordings)**.

Cell property	Control (*n* = 9)	Anti-GAD (*n* = 8)	*P* value
Resting membrane potential	−68 ± 1 mV	−67 ± 1 mV	0.72
Membrane resistance	44 ± 4 MΩ	55 ± 4 MΩ	0.07
Time constant	11.8 ± 0.5 ms	12.6 ± 0.9 ms	0.47
Voltage sag (injection of −1.0 nA)	−5.1 ± 0.9 mV	−7.4 ± 0.9 mV	0.12
AHP (injection of 0.5 nA)	−5.6 ± 0.9 mV	−6.4 ± 1.0 mV	0.57
AP number (injection of 0.5 nA)	5.1 ± 0.6	6.2 ± 0.8	0.28

**Figure 2:**
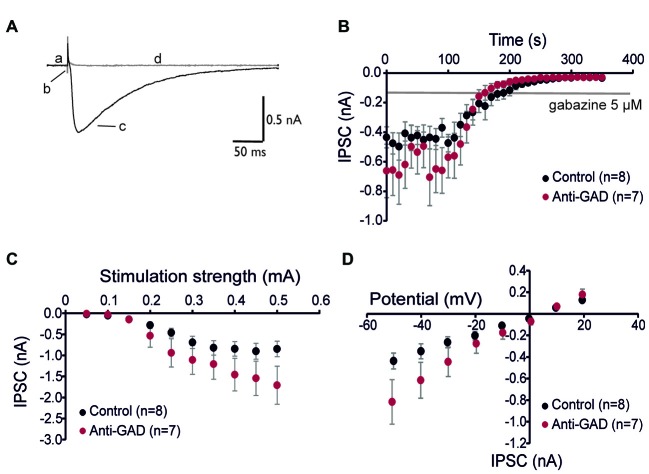
**GABA_A_ receptor-mediated inhibitory postsynaptic currents (IPSCs) are intact in anti-GAD65 tissue. (A)** Superimposed sample traces showing GABA_A_ receptor-dependent IPSCs before (black trace) and after 5 μM gabazine (gray trace). Lowercase letters indicate the current at the holding potential (a), stimulus artifact (b), GABA_A_R-mediated IPSC (c), and stimulation-evoked trace after gabazine (d). **(B)** Whole-cell patch-clamp recordings confirmed that IPSCs evoked by near field stimulation were entirely GABA_A_ receptor-dependent. **(C)** Input-output relationship of GABA_A_R-mediated IPSCs was not significantly different between anti-GAD65 and control tissues. **(D)** The current-voltage relationship revealed that the reversal potential for GABA_A_R-mediated synaptic responses was indistinguishable between anti-GAD65-treated animals and controls.

Since subtle anti-GAD mediated changes in GABA_A_R kinetics can be missed in sharp intracellular recordings, we next performed whole-cell patch-clamp recordings from CA1 pyramidal cells. GABA_A_R-mediated IPSCs were isolated by adding D-AP5 (5 μM) and CNQX (10 μM) and using an almost K^+^−free (0.2 mM) intracellular solution. Figure 2A shows a representative trace of an isolated GABA_A_R-mediated IPSC which was abolished by application of gabazine indicating GABA_A_R-dependence (5 μM, superimposed gray trace in Figure 2A). Importantly, there was no difference in gabazine sensitivity between anti-GAD65-treated and control tissue (Figure 2B). Increasing stimulation strength raised IPSC amplitudes, and the input-output relationship under voltage-clamp conditions confirmed the IPSP data obtained with sharp microelectrodes (Figure 2C). Moreover, the reversal potential of GABA_A_R-mediated IPSCs was indistinguishable between anti-GAD65-treated animals and controls (Figure 2D). It is important to note that with respect to GABA_A_R-mediated IPSC kinetics, no significant difference could be observed (τ_rise_, τ_decay_, IPSC width at 10%, Table 2). Unexpectedly, GABA_A_R-mediated IPSCs actually tended to be larger in anti-GAD-treated tissue compared to controls (Figures 2B–D). Indeed, the charge transfer was doubled in cells from anti-GAD65-treated animals, although this difference was not statistically different (Table 2). Hence, our patch-clamp analyses of CA1 neurons confirmed that neither amplitudes nor kinetics of GABAergic transmission were changed in anti-GAD65-treated tissue compared to control tissue.

**Table 2:** **Kinetic properties of GABA_A_-receptor mediated IPSCs (patch-clamp recordings)**.

Kinetic property	Control (*n* = 7)	Anti-GAD (*n* = 7)	*P* value
Rise time constant (τ_rise_)	3.6 ± 0.6 ms	4.0 ± 0.7 ms	0.76
Decay time constant (τ_decay_)	69.0 ± 7.3 ms	69.9 ± 1.2 ms	0.96
IPSC width at 10%	185.8 ± 17.5 ms	185.4 ± 26.2 ms	0.99
Latency	3.3 ± 0.3 ms	3.2 ± 0.3 ms	0.80
Charge (AUC)	−42.6 ± 10.4 pC	−87.1 ± 28.9 pC	0.20
Coefficient of variation	4.7 ± 0.9 %	4.1 ± 0.8 %	0.51

### Spontaneous GABAergic IPSCs Are not Altered in Anti-GAD65 Tissue

**Figure 3:**
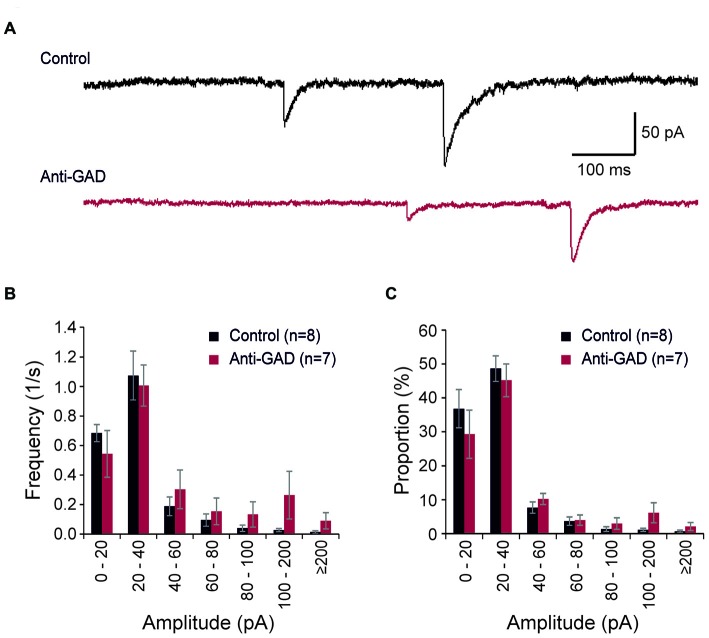
**Spontaneous IPSCs are not altered in anti-GAD65 tissue. (A)** Sample traces of spontaneous IPSCs in control and anti-GAD65 tissue. **(B)** Histogram showing the frequencies of distinct sIPSC amplitude classes revealing no difference in distribution. **(C)** Histogram showing the relative proportion of sIPSC amplitude classes revealing again no difference in distribution.

We recorded also spontaneous inhibitory postsynaptic currents (sIPSCs) in our patch-clamp experiments (Figure 3A). These sIPSCs were completely blocked by gabazine (data not shown). In total, we detected 643 sIPSCs in 306 s in control tissue (8 cells) and 802 sIPSCs in 327 s in anti-GAD65 tissue (7 cells). On average, the sIPSC frequencies were not significantly different between control (2.1 ± 0.3 Hz, *n* = 8 cells) and anti-GAD65 tissue (2.5 ± 0.6 Hz, *n* = 7 cells). In addition, no significant difference was obtained in sIPSC amplitudes (Control: 30.2 ± 2.5 pA, *n* = 8; Anti-GAD: 41.5 ± 8.7 pA, *n* = 7). Since calculating the mean frequency and the mean amplitude might underestimate changes in the distribution of sIPSCs, we next plotted histograms showing the frequencies and relative proportions of sIPSCs in distinct classes of amplitudes (Figures 3B,C). However, statistical evaluation using two-way ANOVA only revealed significant differences between amplitude classes (*P* < 0.05), but no significant treatment effect (i.e., Control vs. Anti-GAD). Hence, spontaneous IPSCs were not altered in anti-GAD65 tissue as assessed by the mean frequency, the mean amplitude and their distribution.

## Discussion

Antibodies against GAD of 65 kDa (anti-GAD65) in the CSF have been described in patients with SPS (Solimena et al., 1988), but also in a subgroup of patients with late-onset isolated cerebellar ataxia (Honnorat et al., 2001) or limbic encephalitis and epilepsy (Peltola et al., 2000; Saiz et al., 2008; Malter et al., 2010). Since GAD65 is the key enzyme for GABA synthesis in the CNS, the pathophysiological role of these antibodies has commonly been ascribed to an inhibition of GABA synthesis and thus GABAergic transmission (Dinkel et al., 1998). The present study used *in vivo* stereotactic injection of human anti-GAD-containing CSF into rat hippocampus. However, GABAergic transmission remained intact in these slices arguing against GABA depletion in anti-GAD65 treated animals.

What is the pathophysiological role of anti-GAD? Following the demonstration that anti-GAD interacted with GABAergic neurons, Dinkel et al. (1998) demonstrated that IgG in the sera of SPS patients reduced GAD activity in crude rat cerebellar extracts, leading to reduction in GABA synthesis. However, the enzymatic activity was preserved when purified IgG from anti-GAD-positive diabetic patients was used suggesting differential epitope specificity of anti-GAD distinguishing neurological from diabetic cases (Dinkel et al., 1998; Ishida et al., 1999; Manto et al., 2007). In addition, electrophysiological data confirmed this view, because IgG from an anti-GAD-positive patient with ataxia suppressed inhibitory transmission onto cerebellar Purkinje cells (Ishida et al., 1999; Mitoma et al., 2000, 2003). Similar results were subsequently obtained in a patient with progressive cerebellar ataxia, unresponsive to intravenous administration of immunoglobulins, suggesting an irreversible damage to cerebellar neurotransmission (Takenoshita et al., 2001). These data suggest that—at least in cerebellar ataxia—down-regulated GABA synthesis might disinhibit Purkinje cells and thus contribute to the manifestation of the disease. There is only one report on hippocampal neurons in culture, where anti-GAD-positive sera were reported to interfere with GABA function and consequently with neuronal inhibition (Vianello et al., 2008). The pathophysiological role of anti-GAD in epileptic seizures has not been addressed systematically before. However, it is obvious that a relevant role of anti-GAD65 should imply a dysfunction of GABAergic neurotransmission. In the present study, intracellular as well as patch-clamp recordings from CA1 neurons failed to detect amplitude or kinetic changes of evoked GABA responses. Interestingly, in parallel to our investigation, similar results were obtained in another group using hippocampal slices cultures. In this preparation, incubation with serum from an anti-GAD65 patient had no effect on evoked or spontaneous GABAergic transmission (Stemmler et al., 2015). Thus, dysfunction of phasic GABAergic transmission does not play a major role in governing epileptogenicity. This is in contrast to the data of Ishida et al. (1999) studying cerebellar ataxia and can be viewed as a strong argument against reduced GABA synthesis in anti-GAD65 encephalitis-associated epilepsy.

What might be the pathophysiological role of anti-GAD65 in epilepsy? In the present study, we have largely obtained negative results using human CSF samples containing anti-GAD65. At least, we have observed that the membrane resistance of CA1 neurons in anti-GAD65 tissue was substantially higher than in control tissue. This implies a stronger voltage deflection in response to transmembrane currents and may lead to impaired shunting inhibition. On the other hand, gabazine depolarized cells from anti-GAD65 tissue significantly stronger than control cells arguing against this idea. Given the majority of negative data, one might argue that we cannot be sure that the antibodies have properly reached sufficient concentrations in the tissue. Alternatively, the injected antibodies may have been cleared from the tissue. Although we cannot entirely exclude these issues, we believe that this is unlikely to be the case, because we have conducted a separate study on anti-NMDAR CSF in parallel to the experiments described here. By using undiluted CSF samples from anti-NMDA receptor encephalitis patients, we could demonstrate an NMDA receptor-specific phenotype including impaired NMDA receptor-dependent transmission and synaptic plasticity (Würdemann et al., 2016). Moreover, impairment of NMDA receptor function was preserved for up to 10 days after surgery. In addition, we also found differences in the Morris water maze hidden platform task indicating an altered hippocampus-dependent spatial learning and suggesting that a single stereotactic injection of CSF can exert behavioral effects for up to 2 weeks (Würdemann et al., 2016). This study demonstrated that diffusion or clearance of antibodies in the hippocampal parenchyma appears to occur on a very slow timescale. Since we used undiluted CSF samples in these experiments as in the present study, we believe that the negative results obtained here do suggest that anti-GAD65 has no effect on GABAergic synaptic transmission which is in line with previous reports (Stemmler et al., 2015; Werner et al., 2015).

Nevertheless, there is at least one alternative explanation of anti-GAD65 effects on neurons, again derived from data on SPS or cerebellar ataxia. Using a combined microdialysis and electromyography approach, Manto et al. (2007) showed that anti-GAD65 decreased the NMDA receptor-mediated NO production and thus impaired the synaptic regulation of glutamatergic transmission. At cerebellar synapses, NO inhibits the presynaptic glutamate release, and anti-GAD65 hence resulted in increased concentrations of glutamate in deep cerebellar nuclei. On the functional level, anti-GAD65 administration *in vivo* caused continuous motor activity with repetitive discharges, abnormal exteroceptive reflexes, and increased excitability of anterior horn neurons (Manto et al., 2007).

All in all, the pathophysiological role of anti-GAD65 is not unequivocal. One major argument against a direct role is that GAD65 is an intracytoplasmic enzyme both in neuronal and pancreatic cells, which is not exposed on the cytoplasmic membrane during GABA release. Under certain circumstances at least, antibodies may reach intracellular targets. While ability of IgG to enter neurons has already been mentioned more than 20 years ago (Fishman et al., 1990, 1991; Yanase et al., 1994), only recent work has demonstrated that IgG can be internalized by neurons, making intracellular antigens accessible in experimental settings (Geis et al., 2010). Hence, albeit antibodies may reach intracellular targets, it appears to be a rare phenomenon and it is unclear whether this mechanism is also relevant in the *in vivo* condition. An alternative explanation could be that anti-GAD65 antibodies cross-react with another, yet unknown target, which is approachable on the neuronal surface, such a mechanism has been described for anti-nuclear antibodies (Madaio et al., 1996). Furthermore, additional antibodies might coexist with the initially identified antibodies. At least in SPS, additional antibodies such as anti-GABA_B_-receptor have been described (Lancaster et al., 2010; Boronat et al., 2011). Importantly, in the presence of IgG from SPS GABAergic transmission and presynaptic GABAergic vesicle pool size were preserved (Werner et al., 2015). A further argument against a pure GAD-inhibiting effect of anti-GAD65 is derived from the diversity of anti-GAD-associated disorders such as neurological and diabetic manifestations. Even more intriguing, but completely unresolved is the fact that some neurological patients with anti-GAD65 develop epileptic seizures, while other suffer from cerebellar ataxia or SPS. This would be difficult to explain if all autoantibodies in these conditions had the same effect—namely blocking GAD enzyme activity.

In summary, we show that CSF containing GAD65-antibodies does not alter GABAergic transmission as proposed for cerebellar manifestations of anti-GAD65 related disorders. Since GAD65-antibodies may show a high variability with respect to their biological effects and pathological sequelae, there is certainly a need to gather further serum or CSF samples in these rare diseases in order to unravel the complex molecular pathophysiology of anti-GAD65 autoimmunity.

## Disclaimer

CGB gave scientific advice to Eisai (Frankfurt, Germany) and UCB (Monheim, Germany), undertook industry-funded travel with support of Eisai (Frankfurt, Germany), UCB (Monheim, Germany), Desitin (Hamburg, Germany), and Grifols (Frankfurt, Germany), obtained honoraria for speaking engagements from Eisai (Frankfurt, Germany), UCB (Monheim, Germany), Desitin (Hamburg, Germany), diamed (Köln, Germany), Fresenius Medical Care (Bad Homburg, Germany), and received research support from Astellas Pharma (München, Germany), Octapharma (Langenfeld, Germany), diamed (Köln, Germany) and Fresenius Medical Care (Bad Homburg, Germany). His employer (Krankenhaus Mara, Bielefeld, Germany) runs a laboratory for the detection of auto-antibodies including those described in this study; external senders are charged for antibody diagnostics.

## Author Contributions

JKH, LM and MR performed experiments and analyzed data. CGB and RK designed the study and wrote the manuscript. CGB provided cerebrospinal fluid samples. TK designed the study, analyzed data and wrote the manuscript. All authors approved the work for publication.

## Conflict of Interest Statement

The authors declare that the research was conducted in the absence of any commercial or financial relationships that could be construed as a potential conflict of interest.
